# Supplementary material for: Cytoskeletal tension actively sustains the migratory T‐cell synaptic contact
Source: EMBO J. 2020 Jan 2;39(5):e102783. doi: 10.15252/embj.2019102783 (PMC7049817; doi:10.15252/embj.2019102783)

Appendix for "Cytoskeletal tension actively sustains the migratory T cell synaptic contact"

Sudha Kumari, Michael Mak, Yehchuin-Poh, Mira Tohme, Nicki Watson, Mariane Melo, Erin Janssen, Michael Dustin, Raif Geha and Darrell J. Irvine.

Table of contents:

1. Appendix Figure legends
2. Appendix Figure S1
3. Appendix Figure S2
4. Appendix Figure S3
5. Appendix Figure S4
6. Appendix Figure S5
7. Appendix Figure S6
8. Appendix Figure S7
9. Appendix Figure S8
10. Appendix Figure S9
11. Appendix Figure S10

Appendix Figure S1. Antigen encounter triggers nucleation of actin foci and cellular symmetry. Related to Figure 1. APS with indicated ligands were incubated with T cells for 2 min, fixed, stained with phalloidin-Alexa488 and imaged using SIM. The graph shows quantification of cell shape in cells. Scale bar, 5µm. P values ***<0.0001, as measured using Mann-Whitney test.

Appendix Figure S2. The aspect ratio (AR) reports on synaptic interface elongation associated with radial interface symmetry breaking. Related to Figure 1. (A) Interference reflection microscopy (IRM) of mouse CD4$^+$ T cells (T cells) shows that significant changes in shape and motility of T cell contact interfaces can be recorded within a time span of 2 min, between their 'arrested' (sedentary) and 'motile' states. The motile or arrested cells were manually identified in the time-lapse images, 20 min post their initial contact with APS, and associated mean aspect ratios and speed were analyzed over a time span of 2 min. The graph shows an average value of sped or AR spanning 2 min. (B) Alteration in speed and shape measured at the population level during synapse breaking. Snapshots of T cells from time-lapse IRM imaging after seeding on APS, with overlaid center-of-mass tracks over time (in color). Shown at right are the speed and aspect ratios calculated within a 2 min window of observation at 5 min or 20 min post cell seeding; points are individual cells. Scale bars, 5µm. P values ***<0.0001, as measured using Mann-Whitney test.

Appendix Figure S3. Calcium sequestration using BAPTA does not predispose cells to synapse breaking. Related to Figure 1. T cells were incubated with APS for 5 min or for 20 min, along with vehicle control or BAPTA and

EGTA in the last 10 min of incubation, fixed, stained with phalloidin-Alexa568 and anti-Talin antibody, imaged using SIM (A). Note that the BAPTA-treated cells retain symmetry more than the control cells, and display significantly more foci, even when they have comparable talin recruitment at the synapse (B). P values; **≤0.005, n.s. for talin= 0.067; n.s. for total actin= 0.22. P values for the comparisons not shown >0.05 as measured using Mann-Whitney test. The points in the plots are the values obtained from individual cells normalized to mean of 5' values. Scale bar, 5µm.

Appendix Figure S4. Synapse symmetry breaking in T cells activated using BMDCs. OTII T cells were activated on OVA peptide-loaded BMDCs for the indicated time points, fixed, stained and imaged using spinning disc confocal microscopy. The images show maximum intensity projection of the area encompassing T cell-BMDC contacts (A), the arrows in left panels show synapse sites, and the arrows in right panels show foci sites within a synapse. P values in quantifications in (B), ***<0.0001; ** in pWASP= 0.006; ** in AR= 0.001; p values for the comparisons not shown >0.05, as measured using Mann-Whitney test. The points in the bottom left plot are the values obtained from individual cells normalized to mean of 5' values. (C) Shows the actin foci in a 5' synapse formed at the pericellular edge of BMDC. Scale bars, 5µm.

Appendix Figure S5. (A-B) WASP overexpression restores actin foci and synapse symmetry. (A) Western blotting of endogenous WASP and overexpressed GFP-WASP in T cells after 20 min incubation on anti-CD3/ICAM-1-coated substrates. The numbers in the graphs represent ratios of WASP: actin band intensities, normalized to the control lane. (B) TIRF imaging of wild type and GFP-WASP-overexpressing T cells incubated with anti-CD3/ICAM-1 substrates, for analysis of AR, actin, and pCasL levels. Fluorescence levels normalized to mean values at 5 min; points represent data from individual cells. **, p =0.009; ***, p < 0.0001; for n.s., p <0.05 using Mann-Whitney test. Scale bar, 5µm. (C) Synapse symmetry breaking is associated with loss of foci and active WASP in human primary $CD4^+$ T cells. Freshly isolated human $CD4^+$ T cells were activated using the substrates for indicated durations, fixed, stained and imaged using TIRF microscopy. In the graph, p values *** <0.001; p values for n.s. or the comparisons not shown >0.05, as measured using Mann-Whitney test. The points in the left plot in (C) are the values obtained from individual cells normalized to mean of 5' values in each case.

Appendix Figure S6. Related to Figure 3. (A) SIM imaging of 2 min WASP$^{-/-}$ T cells synapses shows that these cells are able to initially generate radially symmetric ICAM-1 ring in their synapse. Cells were incubated with lipid bilayers reconstituted with anti-CD3 and ICAM1-Cy5, fixed, stained for F-actin and talin and visualized using SIM. Scale bar, 5µm. (B) Integrin hyperactivation does not rescue symmetry defects in WASP$^{-/-}$ T cells. WT or WASP$^{-/-}$ T cells were incubated with APS in the presence or absence of 0.5mM $MnCl_2$ for 5 min, fixed and processed for talin, pCasL and F-actin (phalloidin-Alexa 568) visualization, and imaged using TIRFM. P values ***<0.001; *= 0.01; n.s. >0.05, as measured using Mann-Whitney test.

Appendix Figure S7. Related to Figure 3. Intracellular calcium flux is not enough to revert asymmetry in WASP$^{-/-}$ cells. T cells from WT or WASP$^{-/-}$ mice were incubated with APS in the presence of DMSO or 1µM Thapsigargin (Thapsi) for 5'. The cells were then fixed and processed for talin, pCasL and F-actin (Phalloidin-Alexa 568) visualization. Scale bar, 5µm. Note that while Thapsigargin treatment is unable to restore symmetry in WASP$^{-/-}$ cells (A, B), it induces downregulation of talin in both WT and WASP$^{-/-}$ T cells (quantification in B). P values, ***<0.0001; *= 0.01 and 0.02 respectively, as measured using Mann-Whitney test. The points in the plots are the values obtained from individual cells normalized to mean of 'WT' in each case.

Appendix Figure S8. Related to Figure 3. (A) pCasL-enriched actin foci in antigen-specific cell-cell conjugate setting. BMDCs loaded with OTII peptide were incubated with mouse WT or WASP$^{-/-}$ OTII transgenic CD4^{+}T cells for 5', fixed and processed for SIM imaging. The image shows maximum intensity projection from 2µm depth of the synaptic area of a single T cell, marked by a white box. The graph on the right shows actin foci and F-actin (phalloidin-Alexa568) and pCasL intensity, or AR, measured at the synapse. The points in the plots are the values obtained from individual cells normalized to mean of 'WT' in each case. P values, *= 0.01; **= 0.009; ***<0.0001. (B) Representative Total Internal Reflection Fluorescence microscopy (TIRF) images of primary human CD4^{+} T cells isolated from healthy individuals or WAS patients and activated for 5' on APS. Graph on the right shows quantification of actin foci, pCasL, and cell AR normalized to mean values of control healthy individual cells. P values ***< 0.001, as measured using Mann-Whitney test. Scale bars, 5µm.

Appendix Figure S9. Related to Figure 4. (A) Endogenous myosinII distribution at the synapse in the stable (upper panels) and broken phase (lower panels) of synapse, as visualized using SIM. Arrows shows the location of myosin puncta juxtaposed with the actin foci (derived from phalloidin-Alexa658 images). Note that the local

molecular organization inside foci appear different between our simulations and experiments- simulations show that myosinII localizes at the foci, while the immunostaining here shows that myosinII localizes to the inter-foci areas. Future computational studies involving additional higher order molecular level interactions are needed to determine the origins of the intra-foci architecture. Scale bar, 5µm. (B) F-actin architecture and stresses on a curved surface resemble those on the flat surface. Simulation on a curved surface of radius 9.6µm shows that F-actin localizes in the inter-foci regions, away from the periphery, similar to that on a flat substrate in Figure 4.

Appendix Figure S10. Related to Figure 5. Azido-blebb. treatment itself, in the absence of photoactivation, does not influence symmetry breaking. T cells were allowed to form synapse on APS for 5 min, were then treated with DMSO vehicle (control) or with 5µM Azido-blebb. on the substrate. Cells were then imaged and analyzed as described in Figure 5H.

Appendix Figure S1

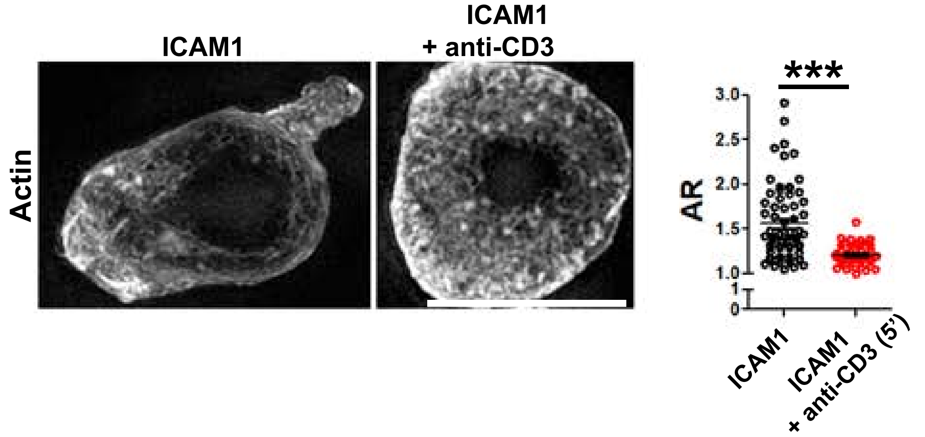

Appendix Figure S2

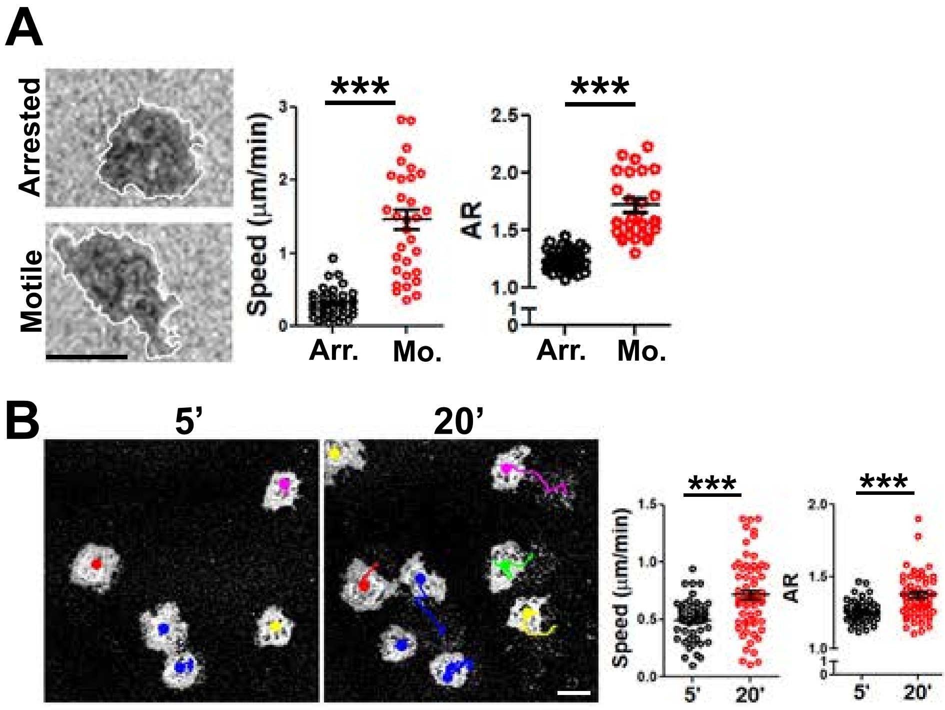

Appendix Figure S3

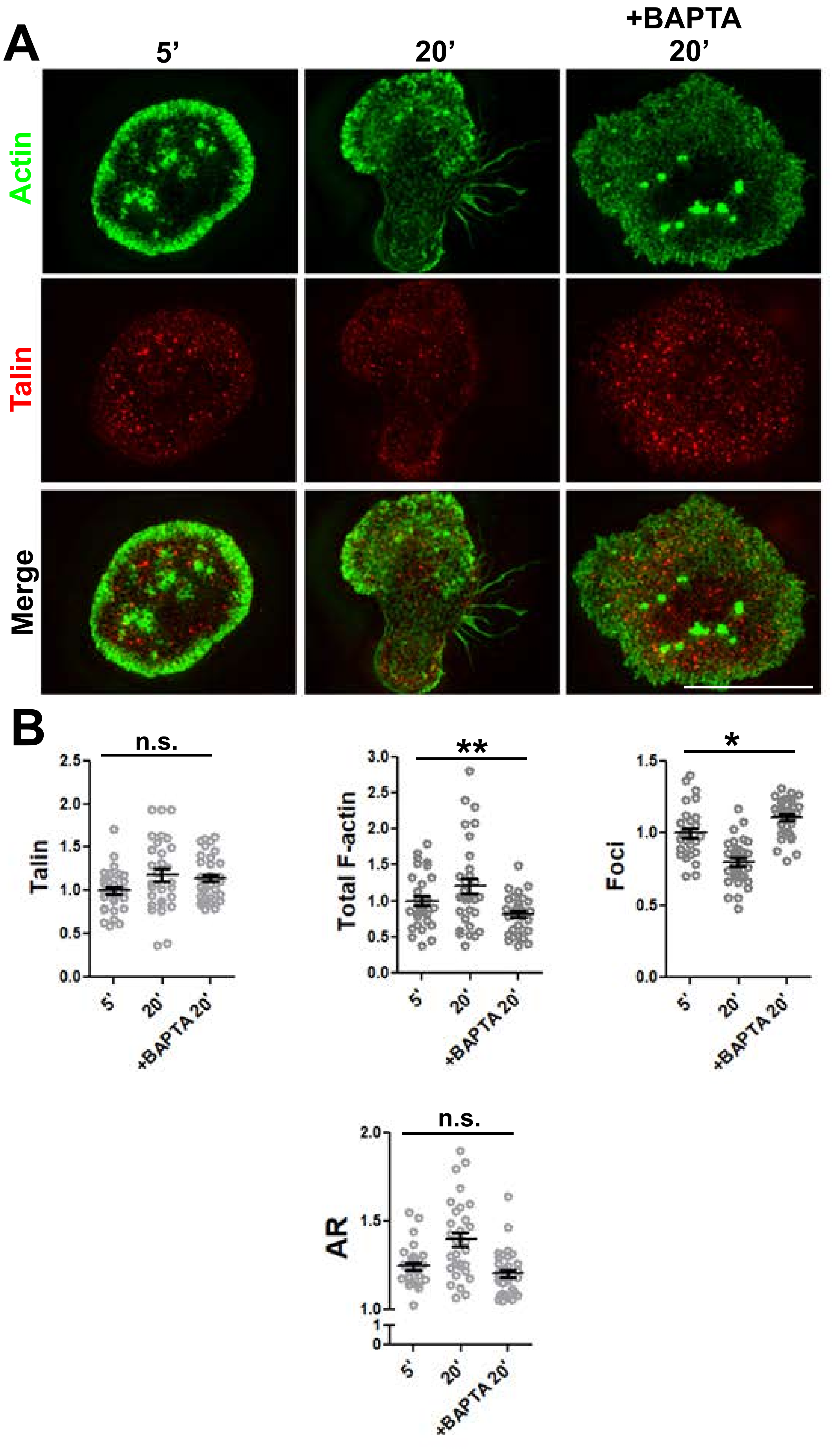

Appendix Figure S4

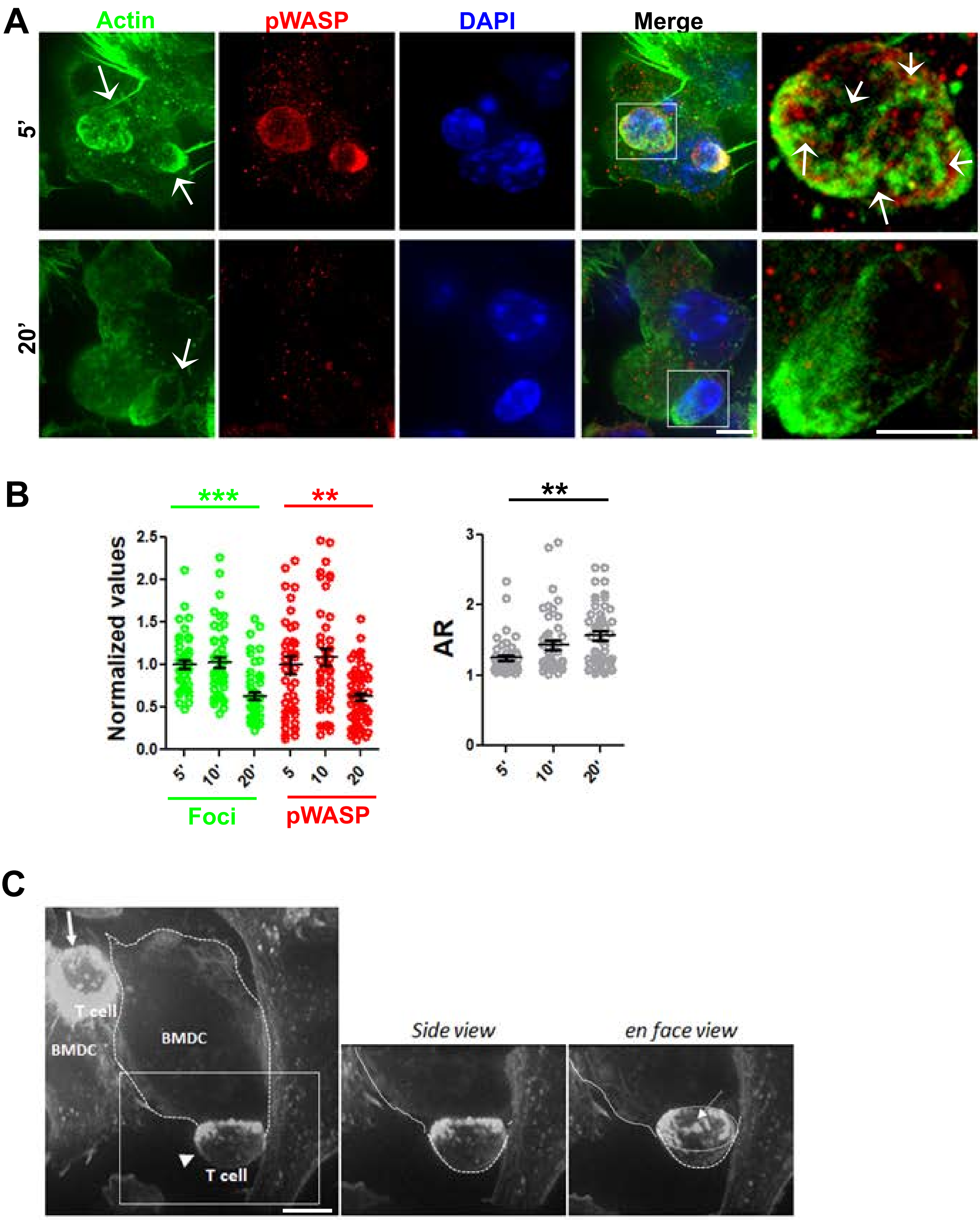

Appendix Figure S5

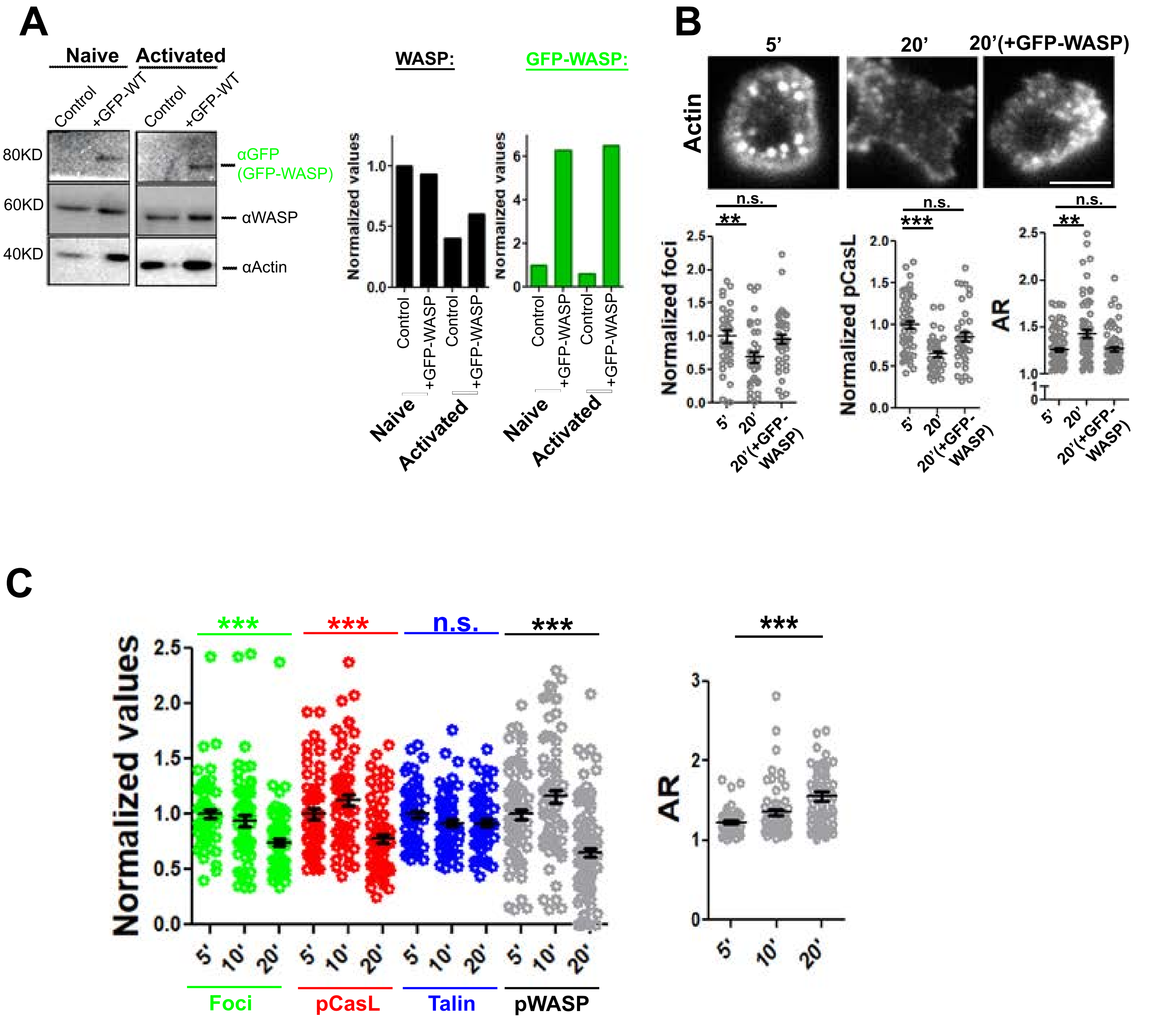

Appendix Figure S6

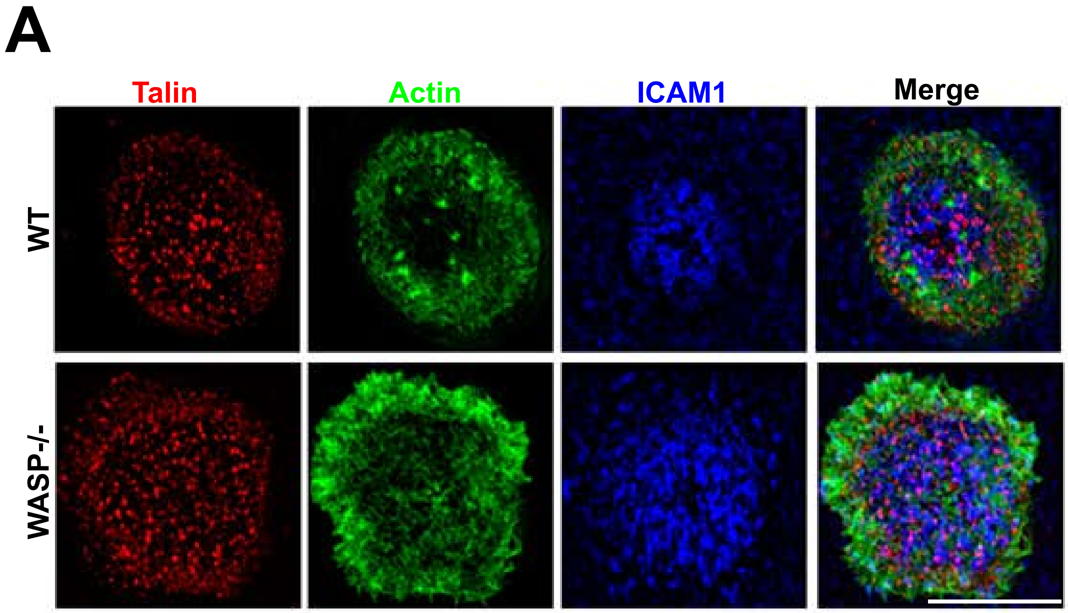

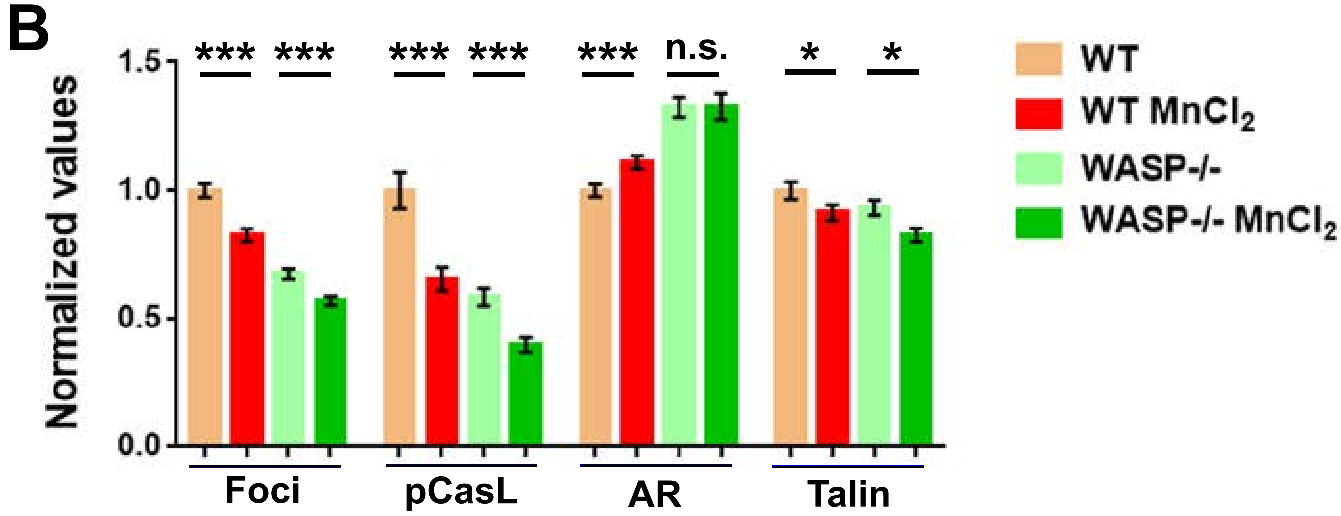

Appendix Figure S7

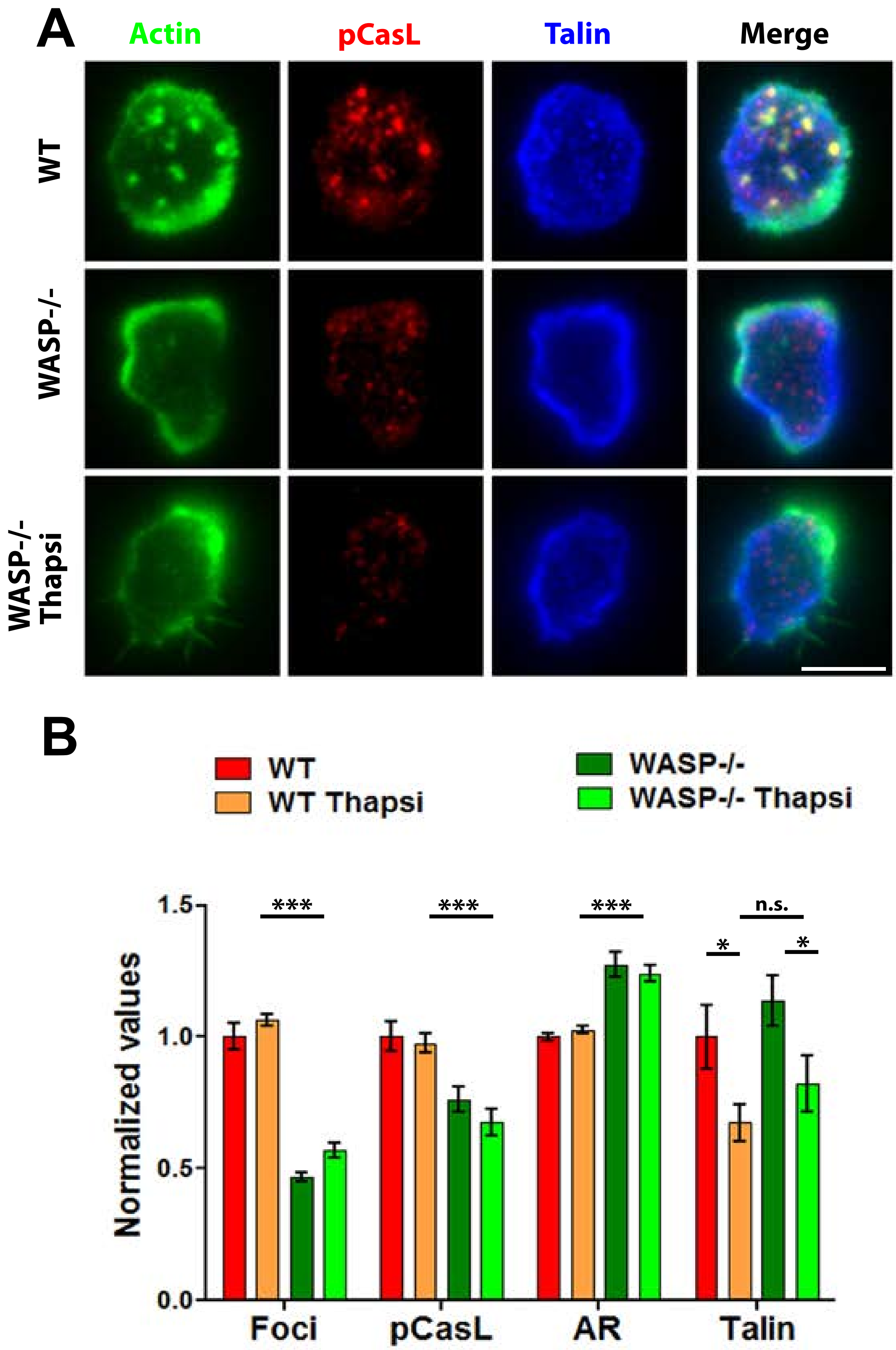

Appendix Figure S8

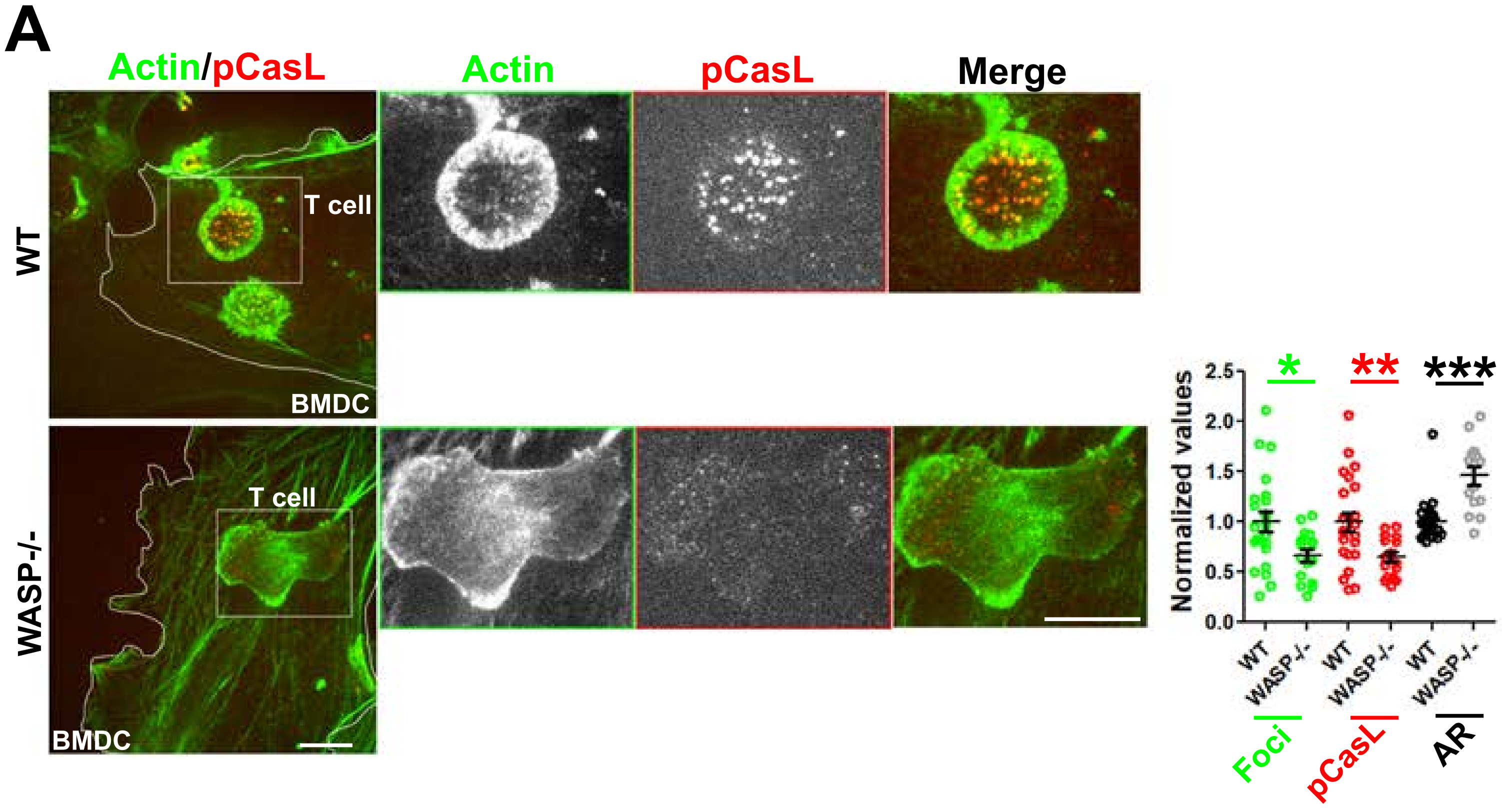

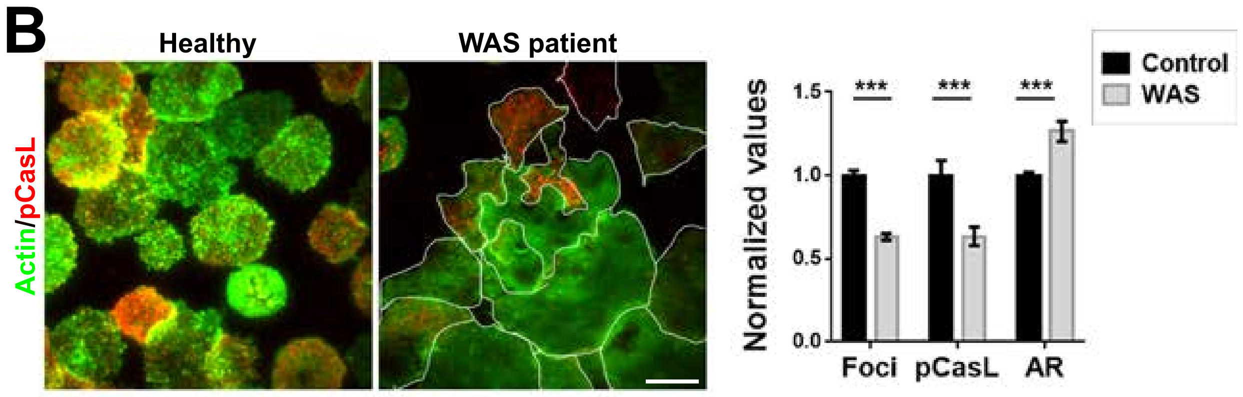

Appendix Figure S9

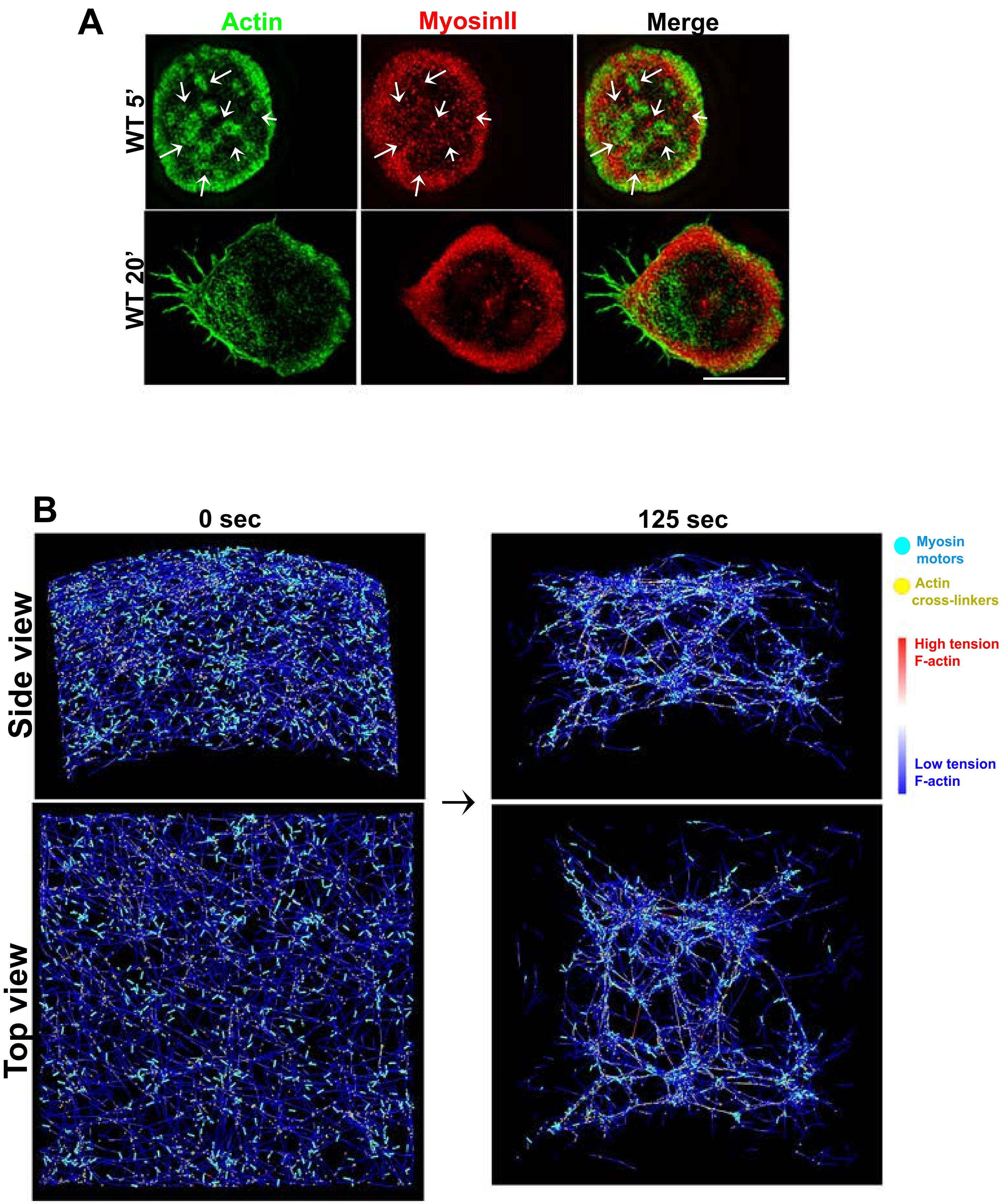

Appendix Figure S10

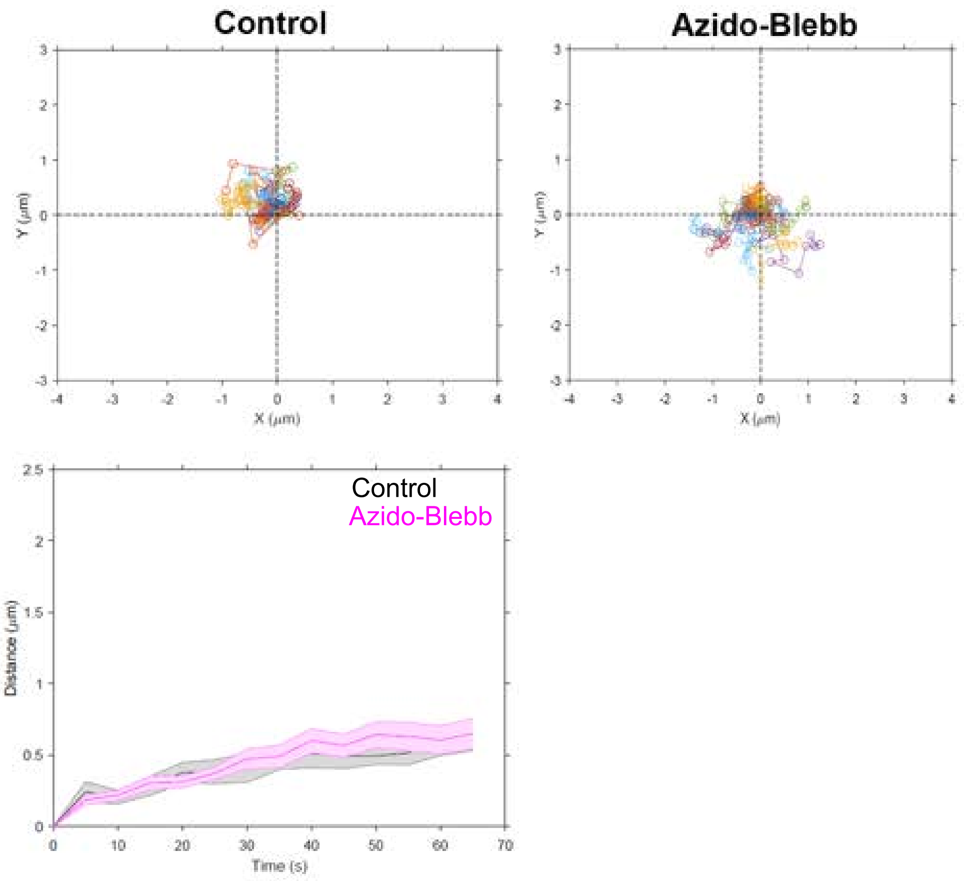

Supplement: Supplementary file 1 — Appendix [file EMBJ-39-e102783-s001.pdf]
