# Supplementary material for: Cytoskeletal tension actively sustains the migratory T‐cell synaptic contact
Source: EMBO J. 2020 Jan 2;39(5):e102783. doi: 10.15252/embj.2019102783 (PMC7049817; doi:10.15252/embj.2019102783)
Supplement: Supplementary file 2 — Expanded View Figures PDF [file EMBJ-39-e102783-s002.pdf]

Expanded View Figures

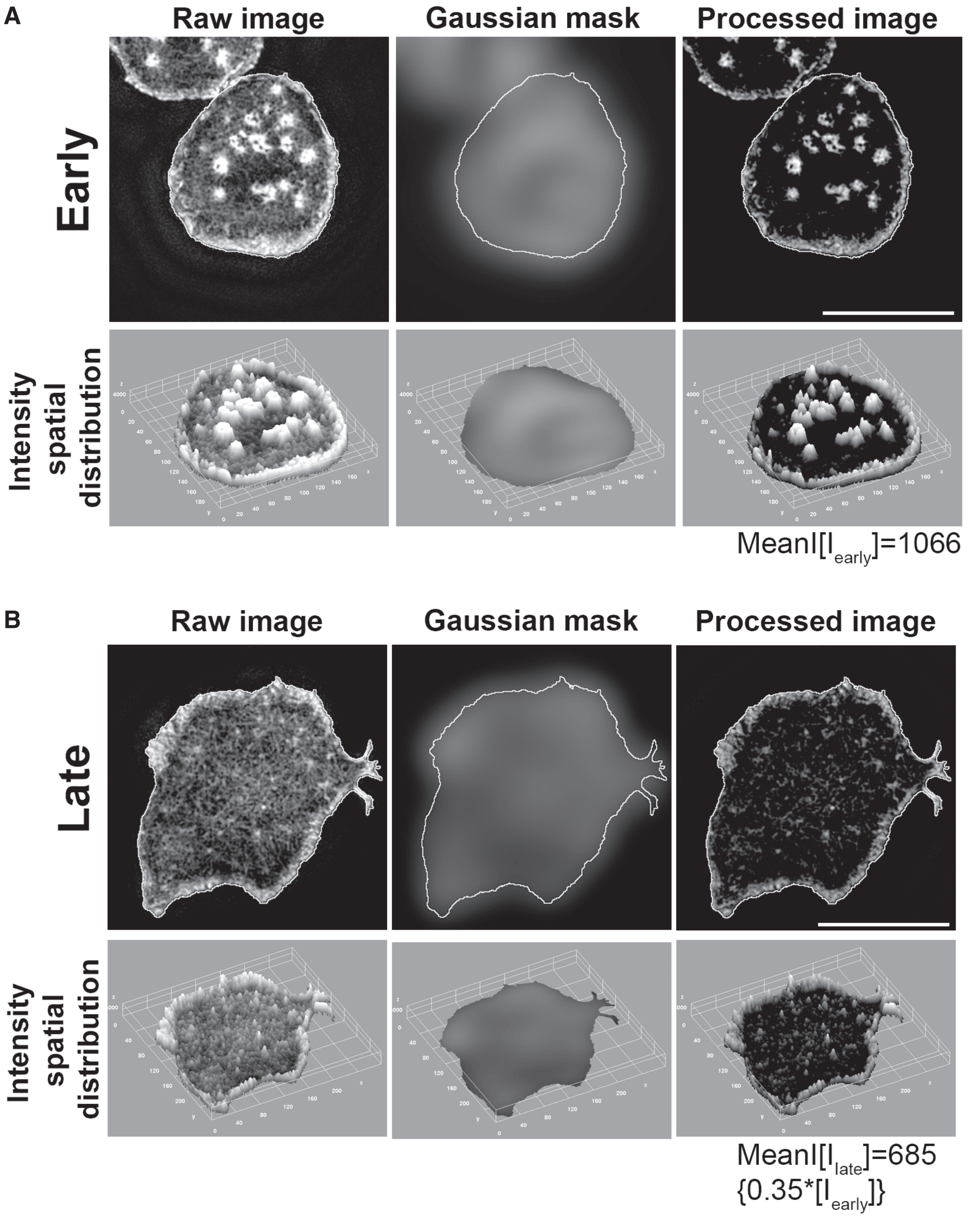

Figure EV1. Image processing scheme utilized to extract and quantify foci on per cell basis. Related to Fig 1.

A, B The images show 2D F-actin intensity as marked by phalloidin staining (top images), or a 3D view of spatial distribution of intensities in the phalloidin images (bottom images). To process the raw images for extracting foci intensities from overall F-actin signal, a Gaussian mask was generated by using a 1.6 × 1.6 μm rolling window, as optimized previously (Kumari *et al*, 2015). Subtraction of mask image from raw image generated a processed image that could be quantified to measure the average intensity contributed by the foci. Note that while this method reliably identifies the foci in raw images and reduces intensity contribution from the non-foci uniform lamellar area, the peripheral lamellipodial network still contributes a background of ~ 35% to the total foci intensity, regardless of the presence of profuse foci in arrested synapse, or their visible reduction in the motile phase. Scale bar, 5 μm.

© 2020 The Authors

Figure EV2. Endogenous pCasL serves as a reliable mechanotransduction marker in T cells. Related to Fig 3.

A A schematic of mechanosensitive CasL phosphorylation in T cells. CasL is recruited to the signaling TCR clusters via LCK via its Src kinase binding domain (SB) and interacts with the F-actin cytoskeleton and adhesion complex binding proteins such as FAK via its SH3 domain. Mechanical tension created due to actin polymerization and actomyosin contraction at the foci sites leads to conformational changes in CasL, exposing tyrosine motifs in its substrate domain. These tyrosine residues are phosphorylated by the local Src family kinases and could be immunolabelled to assess TCR-proximal actin cytoskeletal tension.

B, C Colocalization index of foci and pCasL shows a high degree of association (left plot in C), and correlation with foci intensity per cell (right plot in C). Scale bar, 5 μm.

D pCasL levels are sensitive to broad actin cytoskeletal perturbation. T cells from WT mice were treated with latrunculin A (LatA), or left untreated, during incubation with substrate. Cells were subsequently fixed and processed for immunostaining and imaged using TIRF microscopy. ***$P < 0.0001$, measured using Mann–Whitney test.

E T cells show reduced pCasL in their polarized synapses. **$P = 0.001$, measured using Mann–Whitney test.

F Recruitment of CasL to the synapse is not reduced in WASP$^{-/-}$ T cells. *P* value, ns = 0.58, as measured using Mann–Whitney test. The points in (D–F) are the values obtained from individual cells normalized to mean of WT (D) or 5′ (E) values.

 © 2020 The Authors

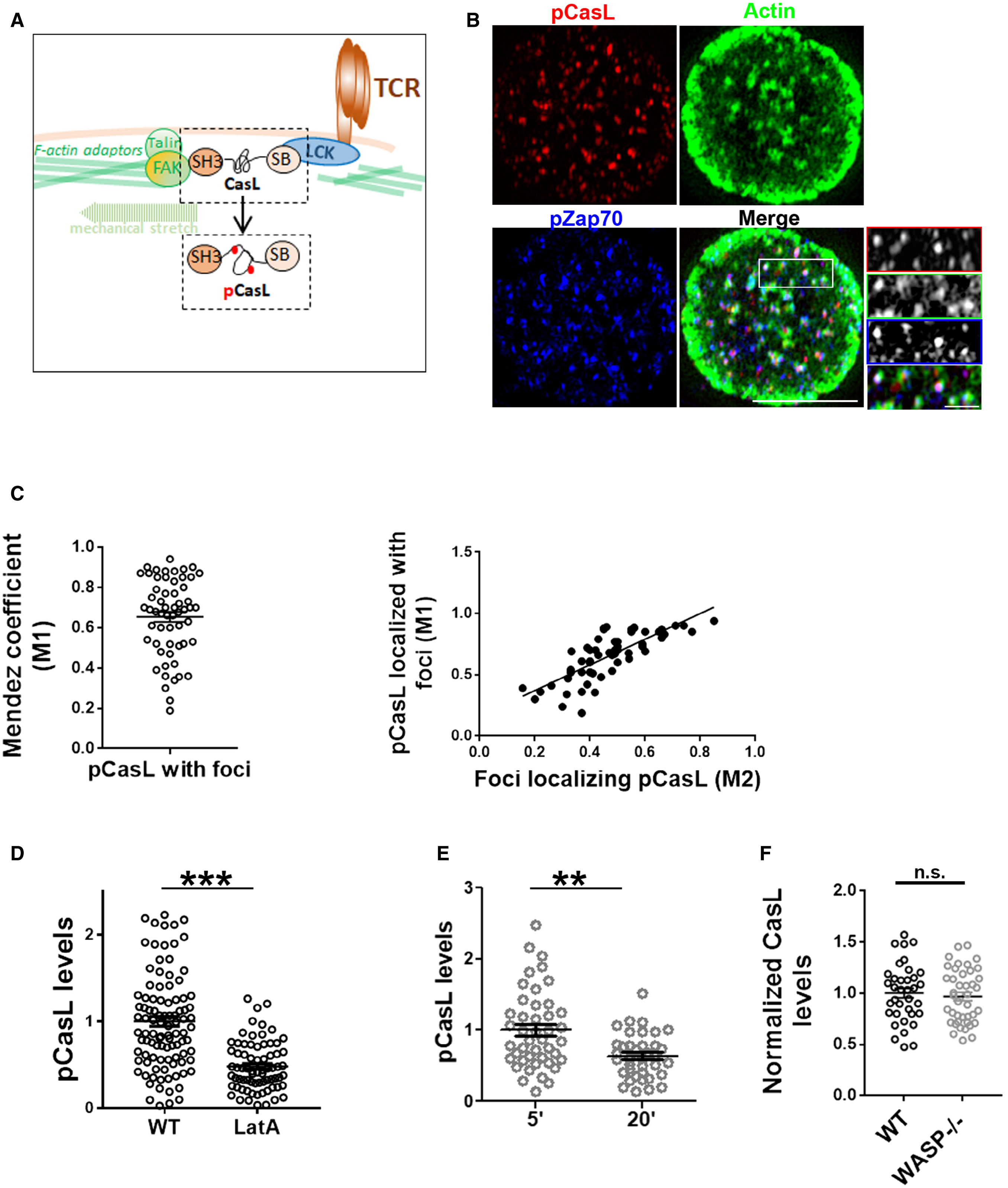
A
TCR
F-actin adaptors
Talin
FAK
SH3
SB
LCK
CasL
mechanical stretch
SH3
SB
pCasL
B
pCasL
Actin
pZap70
Merge
C
Mendez coefficient (M1)
1.0
0.8
0.6
0.4
0.2
0.0
pCasL with foci
pCasL localized with foci (M1)
1.5
1.0
0.5
0.0
0.0 0.2 0.4 0.6 0.8 1.0
Foci localizing pCasL (M2)
D
***
pCasL levels
2
1
0
WT
LatA
E
**
pCasL levels
3
2
1
0
5'
20'
F
n.s.
Normalized CasL levels
2.0
1.5
1.0
0.5
0.0
WT
WASP-/-

Figure EV2.

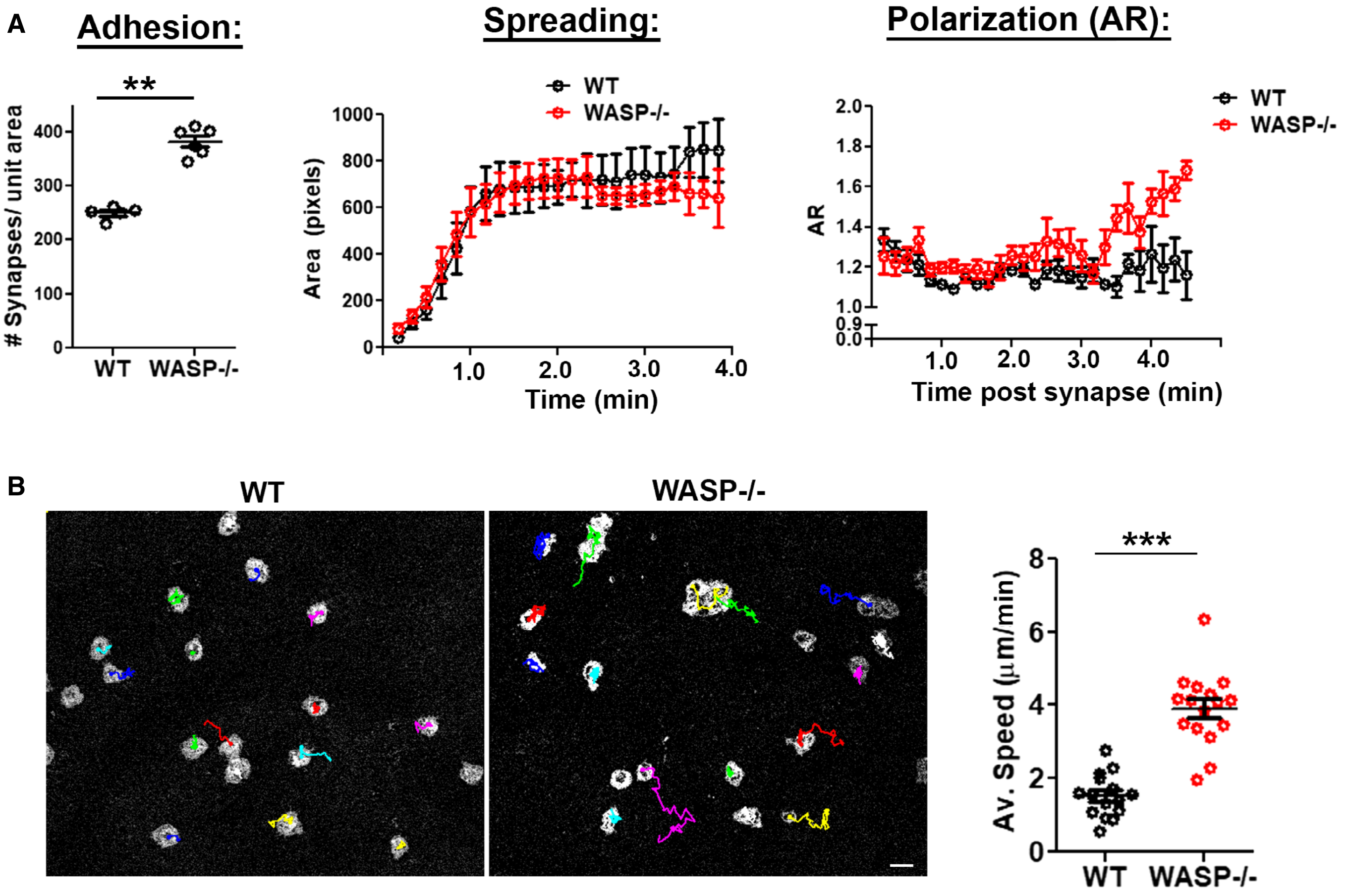

Figure EV3. Comparison of initial adhesion, spreading, and synapse stability in WT and WASP$^{-/-}$ cells. Related to Fig 3.

A Initial adhesion and spreading kinetics are comparable in WASP$^{-/-}$ and WT T cells activated on APS, and WASP$^{-/-}$ cells however polarize their synapse at earlier time point (~ 3 min) than the WT cells, $^{**}P = 0.004$, measured using Mann–Whitney test. T cells were incubated with substrates on a temperature-controlled microscope stage, imaged live using TIRFM (Movie EV5), and analyzed for spreading and shape elongation.

B Synapse breaking in WT and WASP$^{-/-}$ cells. Images are snapshots of T cells, taken from time-lapse IRM images of T cells, and are overlaid with center-of-mass tracks (in color); the graph shows measured speed. $^{***}P < 0.0001$, measured using Mann–Whitney test. Scale bar, 5 μm.

© 2020 The Authors

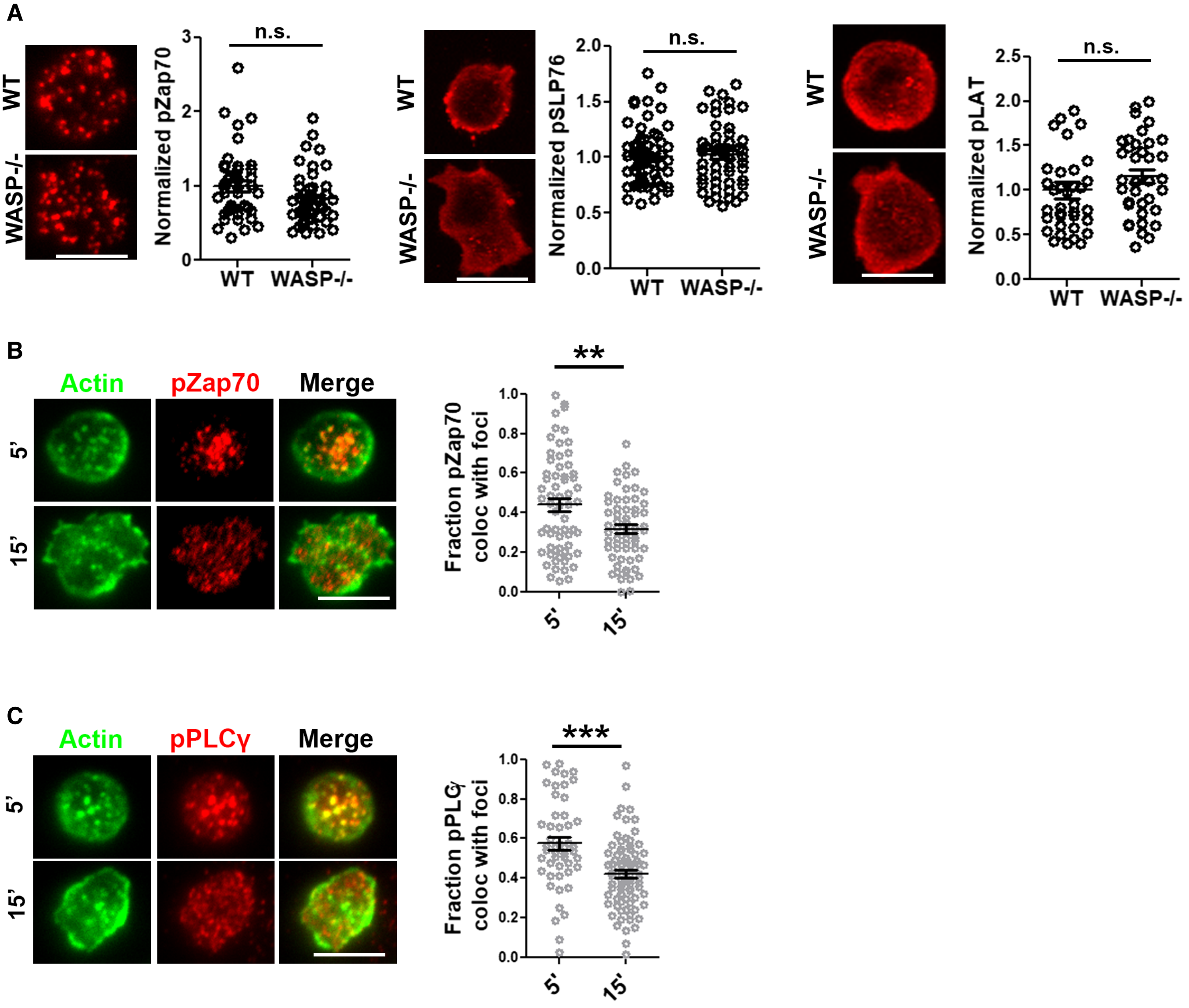

Figure EV4. TCR signaling in WT and WASP$^{-/-}$ cells. Related to Fig 3.

A TCR engagement-induced phosphorylation of early signaling molecules Zap70, SLP76, and LAT. The points in the plots represent values obtained from individual cells normalized to the mean of "WT" values.

B, C Colocalization of foci (derived from Phalloidin—Alexa 568 images) with phospho-Zap70 (B) and with phospho-PLCγ1 (C) is substantially reduced in late (15′) synapses of WT T cells. *P* values n.s. > 0.05; ***< 0.001; **= 0.01, measured using Mann–Whitney test. Scale bars, 5 µm.

Figure EV5. Normal WASP levels and activity are required for synapse stability in human T cells. Related to Fig 3.

A Human CD4$^+$ T cells transduced with control lentivirus or with lentivirus delivering WASP shRNA (as described in Kumari *et al*, 2015, eLife), seeded onto superantigen-loaded HUVEC cells for 5′, fixed, stained for actin (phalloidin-Alexa568) and pCasL, and imaged using a spinning disk confocal microscope. *P* values ***< 0.0001, measured using Mann–Whitney test. The points in top plot are values obtained from individual cells normalized to mean of "control" case.

B Foci polymerization role of WASP underlies its mechanical tension-generating activity. Human CD4$^+$ T cells were transfected with human WT WASP-GFP, WASPΔC, or WASP shRNA (shR)-transducing lentiviral particles. Note that WASP shR and WASPΔC overexpression reduces foci and pCasL at the synapse to a similar extent. The foci values in the graph include background contribution by APC cytoskeletal features underneath the synapse, which are quantified along with foci by our foci extraction algorithm outlined in Fig EV1. The bars in the graph show the mean values normalized to the mean of "WT" in each case. *P* values, *P**** and ** for foci are 0.0003 and 0.004, respectively, and *P*** and *** for pCasL are 0.005 and 0.0004, respectively, measured using Mann–Whitney test. Scale bars, 5 µm.

© 2020 The Authors

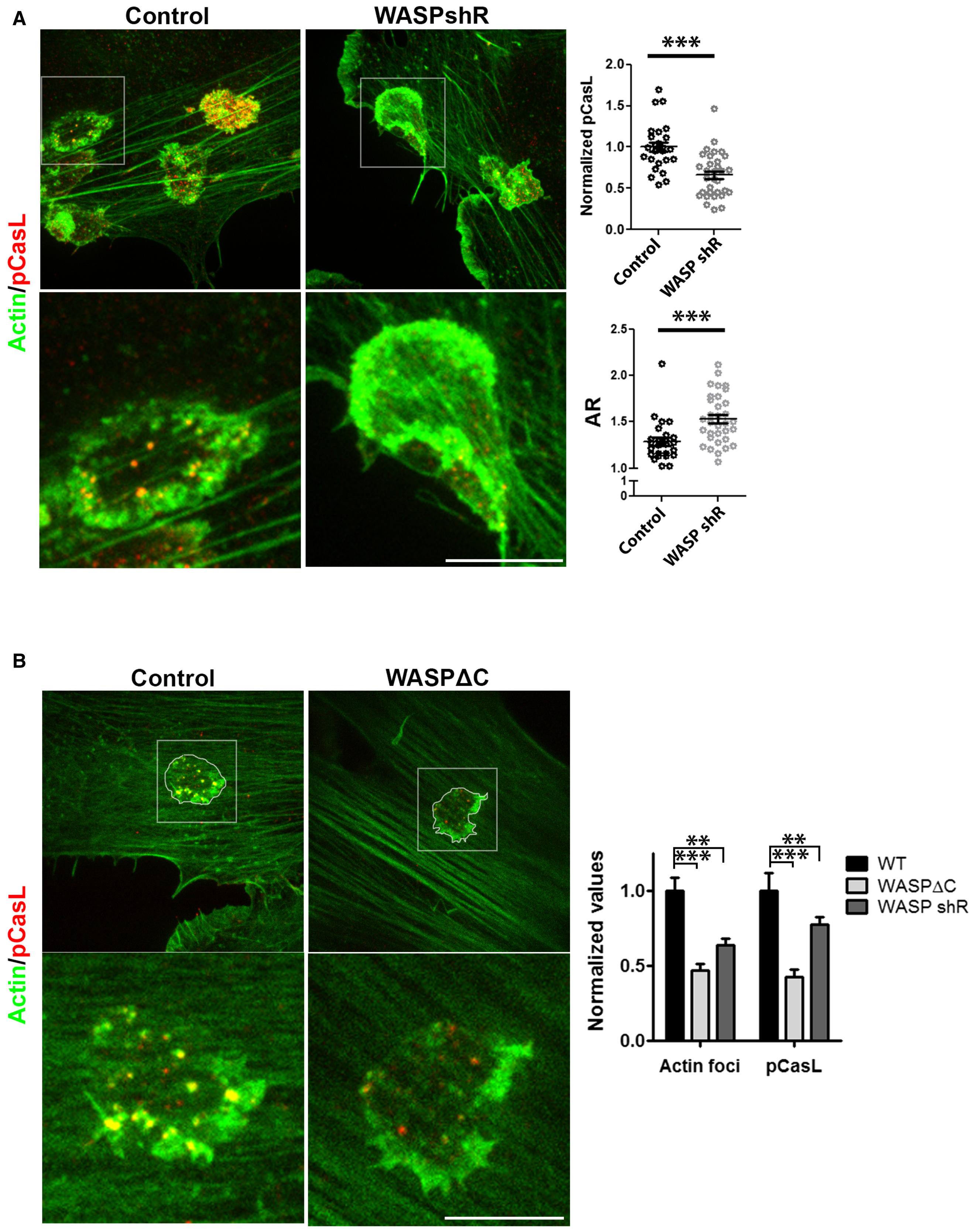

Figure EV5.

© 2020 The Authors
