# Supplementary material for: Cytoskeletal tension actively sustains the migratory T‐cell synaptic contact
Source: EMBO J. 2020 Jan 2;39(5):e102783. doi: 10.15252/embj.2019102783 (PMC7049817; doi:10.15252/embj.2019102783)
Supplement: Supplementary file 3 — Table EV1 [file EMBJ-39-e102783-s003.docx]

**Table EV1. Related to Figure 4:** Model parameters. Parameter values are similar to those used in our previous work where the entire parameter list can be found (*1, 2*). This table shows values important and/or tuned in this study.

| **Symbol** | **Definition** | **Value** |
| --- | --- | --- |
| *C*_A_ | Actin concentration | 25 [μM] |
| % Motors | [Ratio of *C*_M_ to *C*_A_] × 100 | 1 |
| % ACPs | [Ratio of *C*_ACP_ to *C*_A_] × 100 | 1 |
| $k_{\text{u,ACP}}^{0}$ | Zero-force unbinding rate coefficient of ACP | 0.115 [s^-1^](*3*) |
|  | Compliance of a bond for ACP unbinding | 1.04×10^-10^ [m](*3*) |
| *k_a,p_* | Actin polymerization rate | 0.3 [μM^-1^ s^-1^] |
| *k_a,d_* | Actin depolymerization rate | 0.3 [s^-1^] |
| *k_a,n_* | Actin nucleation rate | $n*0.001$ [μM^-1^ s^-1^] |
| *n* | Actin turnover scaling factor | 1, at foci  0.01, at edges |
| *k_adh,unb_* | Zero-force unbinding rate of adhesions between actin and substrate | $n_{adh}*k_{u,ACP}^{0}$ |
| $\lambda_{u,adh}$ | Compliance of a bond for adhesions | $\lambda_{u,ACP}$ |
| *n_adh_* | Adhesion unbinding scaling factor | 0.1, at foci  10, at edges |

Note: The bonds linking actin filaments with actin filaments (ACPs) and actin filaments with the substrate (adhesions) are modeled as slip bonds via Bell’s equation (*4*) with unbinding rates equal to:

$$k_{u}=k_{u}^{0}\exp\left( \frac{\lambda_{u}\left| F \right|}{k_{B}T} \right)$$

where $\left| F \right|$ is the tension acting on the bond, $k_{B}$ is the Boltzmann constant, and $T$ is temperature.

1. M. P. M. Wonyeong Jung, Taeyoon Kim, F-actin cross-linking enhances the stability of force generation in disordered actomyosin networks. *Computational Particle Mechanics* **2**, 317 (2015).

2. M. Mak, M. H. Zaman, R. D. Kamm, T. Kim, Interplay of active processes modulates tension and drives phase transition in self-renewing, motor-driven cytoskeletal networks. *Nature communications* **7**, 10323 (Jan 8, 2016).

3. J. M. Ferrer *et al.*, Measuring molecular rupture forces between single actin filaments and actin-binding proteins. *Proceedings of the National Academy of Sciences of the United States of America* **105**, 9221 (Jul 8, 2008).

4. G. I. Bell, Models for the specific adhesion of cells to cells. *Science* **200**, 618 (May 12, 1978).
