# Supplementary material for: Cytoskeletal tension actively sustains the migratory T‐cell synaptic contact
Source: EMBO J. 2020 Jan 2;39(5):e102783. doi: 10.15252/embj.2019102783 (PMC7049817; doi:10.15252/embj.2019102783)
Supplement: Supplementary file 4 — Movie EV1 [file EMBJ-39-e102783-s004.zip › Movie_EV1/Movie_EV1.docx]

**Movie EV1.** Related to Figure 1. Six different examples of cells breaking their sedentary primary contacts and showing interface shape elongation and a shift in motility, imaged using IRM, indicate that rapid shape transitions can be quantified with 2 min of time duration.
