# Supplementary material for: Cytoskeletal tension actively sustains the migratory T‐cell synaptic contact
Source: EMBO J. 2020 Jan 2;39(5):e102783. doi: 10.15252/embj.2019102783 (PMC7049817; doi:10.15252/embj.2019102783)
Supplement: Supplementary file 5 — Movie EV2 [file EMBJ-39-e102783-s005.zip › Movie_EV2/Movie_EV2.docx]

**Movie EV2.** Related to Figure 1. IRM live imaging of T cells using reveals a significant shift away from the primary synapse site within 20 min of APS encounter. The images were negatively contrasted to identify cell positions and better highlight individual cell boundaries using automated cell outlining (object identification) routine in ImageJ. The residual material left by the T cells on the primary contact site is reminiscent of membrane fragments, as described in Choudhuri et al., 2014.
