# Supplementary material for: Cytoskeletal tension actively sustains the migratory T‐cell synaptic contact
Source: EMBO J. 2020 Jan 2;39(5):e102783. doi: 10.15252/embj.2019102783 (PMC7049817; doi:10.15252/embj.2019102783)
Supplement: Supplementary file 6 — Movie EV3 [file EMBJ-39-e102783-s006.zip › Movie_EV3/Movie_EV3.docx]

**Movie EV3.** Related to Figure 2. LLSM live imaging of mouse T cell synapse expressing LifeAct-GFP, during transition to the motile phase.
