# Supplementary material for: Cytoskeletal tension actively sustains the migratory T‐cell synaptic contact
Source: EMBO J. 2020 Jan 2;39(5):e102783. doi: 10.15252/embj.2019102783 (PMC7049817; doi:10.15252/embj.2019102783)
Supplement: Supplementary file 7 — Movie EV4 [file EMBJ-39-e102783-s007.zip › Movie_EV4/Movie_EV4.docx]

**Movie EV4.** Related to Figure 2. LifeAct-GFP expressing WT or WASP-/- T cells reveal differential dynamics of lamella vs. the foci. Cells were allowed to attach to the APS for 5min (t=0 in the movie), and then imaged using TIRFM.
