# Supplementary material for: Cytoskeletal tension actively sustains the migratory T‐cell synaptic contact
Source: EMBO J. 2020 Jan 2;39(5):e102783. doi: 10.15252/embj.2019102783 (PMC7049817; doi:10.15252/embj.2019102783)
Supplement: Supplementary file 8 — Movie EV5 [file EMBJ-39-e102783-s008.zip › Movie_EV5/Movie_EV5.docx]

**Movie EV5.** Related to Figure 3. WASP-/- cells break contact symmetry faster than the WT T cells. T cells isolated from WT or WASP-/- mice were imaged live using IRM for 10 min at 3 frames/min. The images were negatively contrasted for automated boundary identification (Movie 8; see ‘Methods’) and tracked for the center of mass movement. Bottom panels represent the cell traces overlaid in top panels.
