# Supplementary material for: Cytoskeletal tension actively sustains the migratory T‐cell synaptic contact
Source: EMBO J. 2020 Jan 2;39(5):e102783. doi: 10.15252/embj.2019102783 (PMC7049817; doi:10.15252/embj.2019102783)
Supplement: Supplementary file 10 — Movie EV7 [file EMBJ-39-e102783-s010.zip › Movie_EV7/Movie_EV7.docx]

**Movie EV7.** Related to Figure 4. Simulations showing magnified view of cytoskeletal dynamics around foci in WT synapse.
