# Supplementary material for: Cytoskeletal tension actively sustains the migratory T‐cell synaptic contact
Source: EMBO J. 2020 Jan 2;39(5):e102783. doi: 10.15252/embj.2019102783 (PMC7049817; doi:10.15252/embj.2019102783)
Supplement: Supplementary file 11 — Movie EV8 [file EMBJ-39-e102783-s011.zip › Movie_EV8/Movie_EV8.docx]

**Movie EV8.** Related to Figure 4. Simulations showing evolution of F-actin network in the WASP-/- synapse that is predisposed to breaking. The movie represents a synapse as a rectangular simulation space, based on the scheme presented in Figure 4A.
