# Supplementary material for: Cytoskeletal tension actively sustains the migratory T‐cell synaptic contact
Source: EMBO J. 2020 Jan 2;39(5):e102783. doi: 10.15252/embj.2019102783 (PMC7049817; doi:10.15252/embj.2019102783)
Supplement: Supplementary file 13 — Movie EV10 [file EMBJ-39-e102783-s013.zip › Movie_EV10/Movie_EV10.docx]

**Movie EV10.** Related to Figure 5. LLSM imaging of cells treated with CK666 show a loss of foci and altered actin dynamics in the synapse similar to that in the case of WASP-/- cells.
