# Supplementary material for: Cytoskeletal tension actively sustains the migratory T‐cell synaptic contact
Source: EMBO J. 2020 Jan 2;39(5):e102783. doi: 10.15252/embj.2019102783 (PMC7049817; doi:10.15252/embj.2019102783)
Supplement: Supplementary file 14 — Movie EV11 [file EMBJ-39-e102783-s014.zip › Movie_EV11/Movie_EV11.docx]

**Movie EV11.** Related to Figure 5. Simulation showing the effect of localized myosin perturbation on F-actin network connectivity on the tension within the synapse. The simulation scheme described in Figure 4A was used to create F-actin distribution in synapse, where a perturbation in myosinII was introduced in a rectangular shaped subsynaptic region, shortly after the synapse was established (corresponding to the image shown in Figure 5F).
