# Supplementary material for: Cytoskeletal tension actively sustains the migratory T‐cell synaptic contact
Source: EMBO J. 2020 Jan 2;39(5):e102783. doi: 10.15252/embj.2019102783 (PMC7049817; doi:10.15252/embj.2019102783)
Supplement: Supplementary file 15 — Movie EV12 [file EMBJ-39-e102783-s015.zip › Movie_EV12/Movie_EV12.docx]

**Movie EV12.** Related to Figure 5. Control or Azidoblebb.-treated cells, corresponding to the images shown in Figure 5H (photoactivable inhibition of myosinII).
